# Hematometra and acute abdomen

**DOI:** 10.4103/0974-2700.62117

**Published:** 2010

**Authors:** Ashwini U Nayak, Asha Swarup, Jyothi G S, Sundari N

**Affiliations:** Department of Obstetrics and Gynecology, M.S. Ramaiah Medical College and Teaching Hospital, Bangalore, India

**Keywords:** Hematometra, acute abdomen, ultrasonography

## Abstract

We report a case of a young woman who presented as acute abdomen due to hematometra resulting from cervical fibroid. This uncommon cause of acute abdominal pain should be considered in women especially with amenorrhea.

## INTRODUCTION

The diagnosis of acute abdomen is one of the most daunting tasks in medicine. Acute abdominal pain is the reason for 5% to 10% of all emergency department visits. Hematometra is a rare cause of acute abdomen.

## CASE REPORT

**Figure 1:**
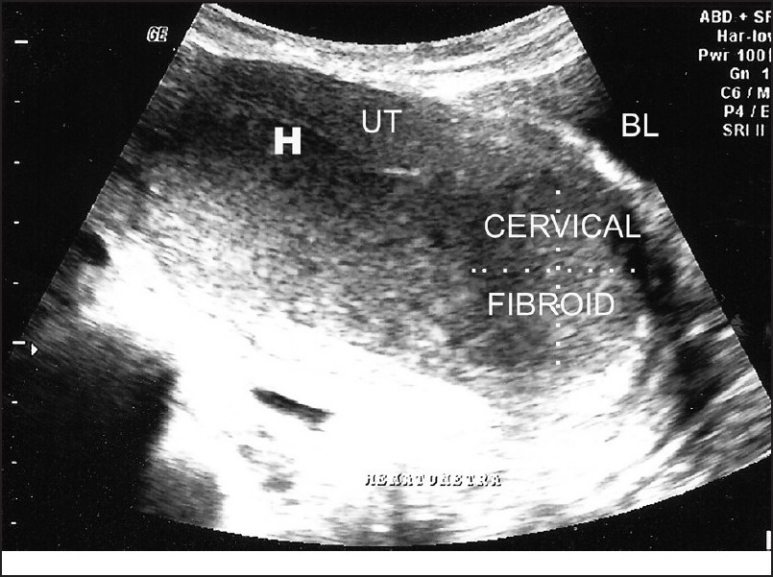
Transabdominal ultrasound revealed an enlarged uterus and cervical fibroid with iso-to hyperechoic collection within the endometrial cavity; BL-bladder, UT-uterus, H-hematometra

**Figure 2:**
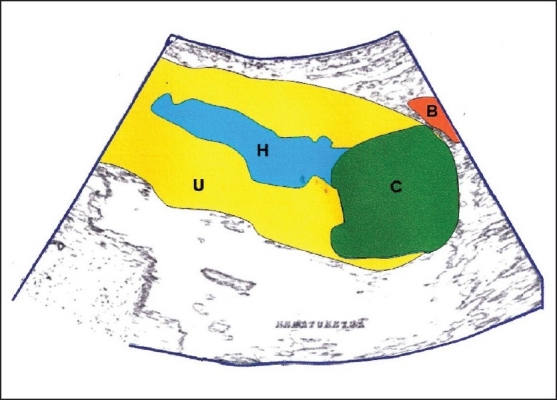
Hand-drawn diagram of the transabdominal scan showing U-uterus; H-hematometra; C-cervical fibroid; and B-bladder

**Figure 3:**
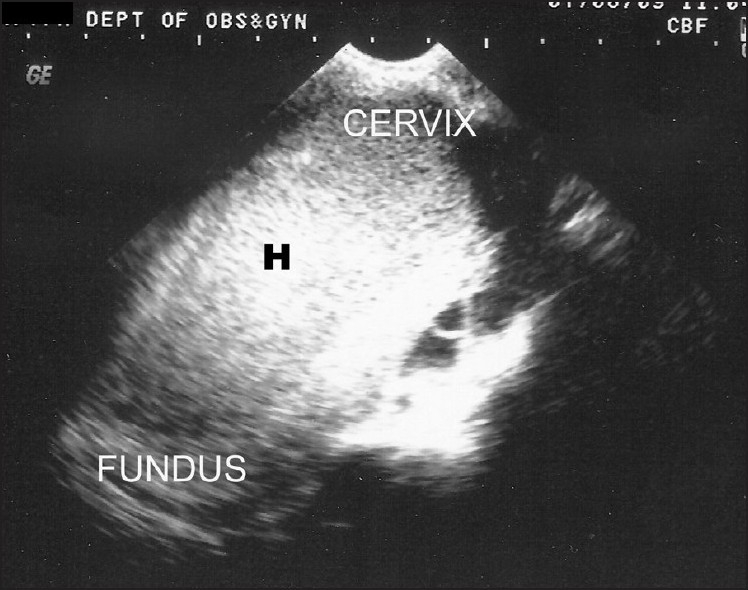
Transvaginal scan showed the iso-to hyperechoic collection in the endometrial cavity clearly; hematometra (H).

**Figure 4:**
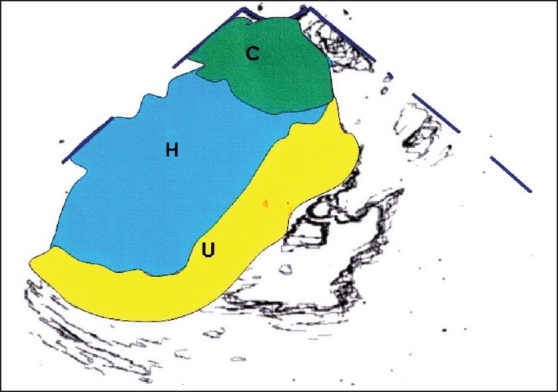
Hand-drawn diagram of the transvaginal scan showing uterus (U), hematometra (H), and cervical fibroid (C)

A 28-year-old female presented as an emergency with 1½ months’ amenorrhea and acute pain abdomen. She had had two normal deliveries and one intrauterine death, which had been followed by manual removal of the placenta. The patient also complained of reduced flow during periods since 2 years. On examination, the vitals were stable. Per abdominal examination revealed tenderness in the hypogastrium and left iliac fossa. A cystic mass of about 7×8 cm size was felt in the left iliac fossa; the lower border of the mass could not be felt. Pelvic examination was limited by pain. Per speculum examination showed a cervical fibroid arising from the posterior lip of the cervix. The os could not be visualized. Per vaginal examination revealed extreme tenderness on movement of the cervix. Movements of the mass were transmitted to the cervix. An emergency transabdominal ultrasound revealed an enlarged hour-glass shaped uterus, with sudden narrowing in the region of the lower uterine segment. There was an iso-to hyperechoic collection within the endometrial cavity with an approximate volume of 100 ml [Figures 1 and 2]. A transvaginal scan showed the collection in the endometrial cavity clearly [Figures 3 and 4]. The urine pregnancy test was negative and routine examination was normal. Hemoglobin was 9 g/l and the white blood cell count 14800/mm^3^. Attempts at dilation of the cervix with Hegar dilators were unsuccessful. The patient underwent diagnostic laparoscopy, which revealed a enlarged cystic uterus. Under anesthesia, the attempt to dilate the cervical os was repeated but was unsuccessful as before.

As the patient had requested for hysterectomy, we decided to proceed with a vaginal hysterectomy. During the procedure, while pushing the bladder, the anterior wall of the thinned-out uterus gave way and thick, old, blood was seen coming out of the rent. Histopathological examination of the specimen showed cervical fibroid. The patient was discharged after an uneventful postoperative period.

## DISCUSSION

Acquired obstruction of the lower female genital tract is rare.[1] Hematometra is a retention of blood in the uterine cavity caused by obstruction to menstrual flow at the level of the uterus, cervix, or vagina. In older women, the obstruction is usually acquired and occurs at the level of the cervix.[2] In young women, hematometra may be due to congenital anomalies such as an imperforate hymen or a noncommunicating Müllerian duct.[3] Transabdominal sonography is a noninvasive imaging modality useful for examining occlusions of the genital tract.[4] Transvaginal sonography is important in the evaluation of hematometra because it affords clear visualization of the endometrial cavity.[5]

## CONCLUSION

The rare possibility of hematometra should be considered in any women presenting with acute abdominal pain, especially when the pain is associated with secondary amenorrhea.
